# Cognitive Distortions Associated with Imagination of the Thin Ideal: Validation of the Thought-Shape Fusion Body Questionnaire (TSF-B)

**DOI:** 10.3389/fpsyg.2017.02194

**Published:** 2017-12-19

**Authors:** Andrea Wyssen, Luka J. Debbeler, Andrea H. Meyer, Jennifer S. Coelho, Nadine Humbel, Kathrin Schuck, Julia Lennertz, Nadine Messerli-Bürgy, Esther Biedert, Stephan N. Trier, Bettina Isenschmid, Gabriella Milos, Katherina Whinyates, Silvia Schneider, Simone Munsch

**Affiliations:** ^1^Department of Clinical Psychology and Psychotherapy, University of Fribourg, Fribourg, Switzerland; ^2^Department of Psychology, Psychological Assessment and Health Psychology, University of Konstanz, Konstanz, Germany; ^3^Department of Clinical Psychology and Epidemiology, University of Basel, Basel, Switzerland; ^4^Provincial Specialized Eating Disorders Program for Children and Adolescents, British Columbia Children’s Hospital, Vancouver, BC, Canada; ^5^Department of Psychiatry, University of British Columbia, Vancouver, BC, Canada; ^6^Mental Health Research and Treatment Center, Ruhr-University Bochum, Bochum, Germany; ^7^Privatklinik Aadorf, Aadorf, Switzerland; ^8^Kompetenzzentrum für Essverhalten, Adipositas und Psyche Spital Zofingen, Zofingen, Switzerland; ^9^Klinik für Psychiatrie und Psychotherapie, Universitätsspital Zürich, Zürich, Switzerland; ^10^Klinik Schützen Rheinfelden, Rheinfelden, Switzerland

**Keywords:** thought-shape fusion body questionnaire, cognitive distortions, body image, thin ideal, eating disorders, women

## Abstract

Thought-shape fusion (TSF) describes the experience of body-related cognitive distortions associated with eating disorder (ED) pathology. In the laboratory TSF has been activated by thoughts about fattening/forbidden foods and thin ideals. This study aims at validating a questionnaire to assess the trait susceptibility to TSF (i.e., body-related cognitive distortions) associated with the imagination of thin ideals, and developing an adapted version of the original TSF trait questionnaire, the *Thought-Shape Fusion Body Questionnaire* (TSF-B). Healthy control women (HC, *n* = 317) and women diagnosed with subthreshold and clinical EDs (*n* = 243) completed an online-questionnaire. The factor structure of the TSF-B questionnaire was examined using exploratory (EFA) and subsequent confirmatory factor analysis (CFA). EFA pointed to a two-factor solution, confirmed by CFA. Subscale 1 was named *Imagination of thin ideals*, containing five items referring to the imagination of female thin ideals. Subscale 2 was named *Striving for own thin ideal*, with seven items about pursuing/abandoning attempts to reach one’s own thin ideal. The total scale and both subscales showed good convergent validity, excellent reliability, and good ability to discriminate between individuals with subthreshold/clinical EDs and HCs. Results indicate that cognitive distortions are also related to the imagination of thin ideals, and are associated with ED pathology. With two subscales, the TSF-B trait questionnaire appropriately measures this construct. Future studies should clarify whether TSF-B is predictive for the development and course of EDs. Assessing cognitive distortions with the TSF-B questionnaire could improve understanding of EDs and stimulate the development of cognitively oriented interventions. Clinical Trial Registration Number: DRKS-ID: DRKS00005709.

## Introduction

A long tradition of research in clinical psychology has demonstrated the negative influence of thin ideals in the media on body dissatisfaction (BD) and eating disorder (ED) pathology in young women (for an overview see e.g., Levine and Harrison, 2009). Whereas earlier studies proposed a direct negative influence of thin ideals presented in media, recent data underline the role of mediators and moderators of such an effect.

Trindade and Ferreira (2014) demonstrated that the impact of BD and appearance-related social comparison on ED pathology is partially mediated by body-related *thought fusion*. This term describes an individual’s difficulty to distract and distance from thoughts, such as distorted body-related thoughts. In the *fusioned* state, individuals fail to regard thoughts objectively as part of their inner experience. Instead, they identify themselves with the thoughts that have detrimental consequences on their emotions and behaviors (Hayes and Pankey, 2002; Hayes, 2004). Moreover, thought fusion is a cognitive distortion associated with psychological inflexibility, which is an important etiological factor in mental disorders and also in EDs (Merwin et al., 2011). Further, cognitive distortions are known to be related to cognitive features in depression such as inflexibility in thinking, rumination, systematic errors, and depressiveness. In the same vein, ED behaviors, such as binge eating or restrictive eating, often represent inappropriate attempts to cope with negative thoughts and emotions. Accordingly, it has been shown that individuals presenting with high BD, a pronounced tendency to upward social comparison, and a stronger internalization of the thin ideal are more negatively influenced by thin ideals in the media (Ferguson, 2013; Hausenblas et al., 2013).

Daily confrontation with thin ideals in the media might further activate selective attention and distorted cognitions such as *Thought-Shape Fusion* (TSF). Shafran et al. (1999) initially described TSF as thoughts about food, weight and shape that are non-veridical and skewed. TSF was adapted from the concept of “Thought-Action Fusion” (TAF) in obsessive-compulsive disorder (OCD) (Shafran et al., 1996; Rachman and Shafran, 1999). In individuals with OCD, there is a belief that the likelihood of an undesirable event occurring is increased by a negative thought (likelihood TAF) and that these negative thoughts are morally equal to the actual performance of the negative behavior (moral TAF) (Shafran et al., 1996, 1999). In EDs, comparable cognitive distortions have been observed. These cognitions include the belief that the mere thought of eating fattening/forbidden food, gaining weight, not exercising, or discontinuing a diet leads to individuals with EDs reporting feeling fatter and more concerned about weight gain (Coelho et al., 2013). While TSF represents a type of cognitive distortion, it is important to note that it is not equivalent to a delusional belief. People who are highly susceptible to TSF probably realize that merely thinking about eating certain foods cannot influence their body shape or weight, but nevertheless have the worry that this is a possible outcome (Shafran et al., 1999). A questionnaire including three theoretical factors to assess the facets of the TSF concept was developed: *Likelihood* (the mere thought of eating fattening/forbidden food leads to the belief that subsequent weight gain is more likely), *moral* (the thought of eating fattening/forbidden food is morally as wrong as the act of eating), and *feeling* (the thought of eating fattening/forbidden food evokes feelings of fatness) (Shafran et al., 1999).

Although TSF levels are higher in women with EDs, TSF occurs also in healthy women of the general population (Coelho et al., 2013). In line with the findings of Trindade and Ferreira (2014), we found that TSF partially mediates the association between BD and ED pathology in men (Wyssen et al., 2016a) and women (Wyssen, 2015). Apart from questionnaire-based data, it has been shown that TSF can be induced in healthy women (Coelho et al., 2010) in the laboratory, and to a stronger degree in women with EDs (Coelho et al., 2008).

The orientation toward an unrealistic thin ideal is widespread, and represents a more reliable predictor of BD in women than actual BMI (Markey and Markey, 2005; Swami et al., 2010). In previous research of our group, we therefore postulated that in addition to food-related TSF, exposure to thin ideals (or the mere imagination of thin ideals) might similarly activate distorted cognitions about one’s own appearance. Supporting the existence of body-related cognitive distortions, a recent laboratory study of our group found, that the experience of body-related cognitive distortions (TSF-B) can be induced not only by thoughts about food, but also by the imagination of thin ideals. A total of 91 healthy women were exposed to either a fashion magazine (displaying the thin ideal) or a neutral magazine (displaying pictures of landscapes) in a waiting room, followed by a request for participants to think about the pictures they saw in the magazine. The manipulation was successful, in that the group with the fashion magazine reported induction of body-related cognitive distortions (e.g., feelings of fatness). Moreover, results showed a significant decrease of mood and body image satisfaction in the fashion magazine group compared to the control group (Wyssen et al., 2016b). The comparison process of an unrealistic *inner* thin-ideal representation with a preexisting negative representation of one’s own body (see e.g., Vossbeck-Elsebusch et al., 2015), might activate self-ideal discrepancy and lead to the feeling of weight gain. TSF-B is proposed as a body-related cognitive distortion, activated by exposure to thin ideals (either internally or externally), which leads to perceived weight gain and moral wrong-doing.

Assessing cognitive distortions such as TSF provides additional insight into the development and maintenance of EDs. TSF has been proposed to be a component of the over-evaluation of food, weight and shape that is a core etiological mechanism in cognitive-behavioral models of EDs (Coelho et al., 2014). Investigating the specificity and validity of body-related cognitive distortions can improve the specificity of the maintaining mechanisms, and ultimately lead to more focused preventive and therapeutic interventions.

Previous studies have demonstrated high internal consistency and predictive validity in both the original TSF trait questionnaire (Shafran et al., 1999) and in the short version (Coelho et al., 2013). However, the factorial structure of the three content-related components likelihood, moral, and feeling TSF could not be confirmed (Jáuregui Lobera et al., 2012; Coelho et al., 2013). In the present study, the short version of the TSF questionnaire (TSF trait) from Coelho et al. (2013) was modified by the authors (SM, AW, LJD) to assess body-related cognitive distortions related to the imagination of thin ideals (*Thought-Shape Fusion Body Questionnaire;* TSF-B). As with the original measure, TSF-B includes items representing the *TSF Concept* (example item: “I feel fatter after picturing thin women.”) as well as an additional section to detect the *Clinical Impact* of TSF-B (example item: “To what extent do thoughts about your thin ideal interfere with your daily life?”).

The aims of the present study are twofold: First the study explores the factor structure of the newly developed *Thought-Shape Fusion Body Questionnaire* (TSF-B), and investigates whether the psychometric properties of the TSF-B differ between healthy participants and those with ED symptoms (i.e., measurement and structural invariance). Second, we assess convergent validity and test whether TSF-B is associated with ED pathology. The validation of the TSF-B measure includes measurement of convergent validity with food-related TSF, BD, thin-ideal internalization and depressive symptoms. We expect more pronounced levels of TSF-B in individuals with higher levels of BD and those who report pressure to conform to an internalized, socially determined thin ideal. We also predict that distorted cognitions, including TSF-B, will be related to higher levels of depression and depressive rumination.

## Materials and Methods

### Participants and Procedure

We obtained data from 591 women (aged between 16 and 47 years). Altogether, 31 individuals prematurely terminated their study participation and were not included in the analyses (drop-out rate of 5.3%). From the remaining 560 participants, 317 women did not meet the criteria of any DSM-5 (Diagnostic and Statistical Manual of Mental Disorders) diagnosis (APA, 2013) according to the diagnostic interview (Diagnostisches Interview für psychische Störungen, DIPS or Mini-DIPS (short version), adapted for DSM-5; Schneider and Margraf, 2011; Margraf, 2015) and the assessment via the EDE-Q (Eating Disorder Examination Questionnaire, German version Hilbert and Tuschen-Caffier, 2006) [for additional information see Supplementary Table 2 (available online)] and were considered as healthy. A total of 243 participants fulfilled the diagnostic criteria of an ED according to DSM-5 on a clinical (67 patients met criteria for AN, 53 patients for BN, 30 patients for Binge-Eating Disorder (BED)), or subthreshold level (e.g., subthreshold BN and BED according to DSM-5 criteria; *n* = 93). For more detailed information on the diagnostic criteria, refer to Supplementary Table 2 (available online). The majority (70%) of participants reported receiving treatment at the time of the study (medical, psychotherapeutical or combined treatment). Recruitment of participants took place from November 2013 to April 2016. HC participants were recruited at the collaborating universities. In addition, online recruitment included different web-based platforms (e.g., self-help groups for individuals affected by ED symptoms), and patients were recruited in several collaborating clinical centers [for more information on studies and recruitment refer to Supplementary Table 1 (available online)]. All patients were enrolled in a parallel research project on the role of TSF in media exposure carried out by two of the authors (SM and SS; see Munsch, 2014). The recruitment and instruction was coordinated and monitored by AW.

Inclusion criteria for both groups (HC and ED) were based on diagnoses assessed with a diagnostic interview (DIPS; Schneider and Margraf, 2011) or its short version (Mini-DIPS; Margraf, 2015). Participants with AN, BN or BED, as well as participants with subthreshold ED diagnoses were included in the transdiagnostic ED group. For the HC group, an inclusion criterion was the absence of any mental disorder according to the diagnostic interview. Additional exclusion criteria for overall study participation were psychotic disorders and serious medical conditions having an effect on eating and mood (such as cancer or diabetes). Individuals were asked to report current prescription medication. All participants were fluent in German.

Participants signed an informed consent prior to participation. The local ethic committees (University of Fribourg, Cantons of Fribourg, Thurgau, Zürich and Aargau, as well as the University of Bochum) approved the study protocols of all studies from which data were included for the present analyses. Most participants were Swiss or German. Of the sample 23% held a high school degree, 50% had a higher education entrance qualification and 21% held a degree from a professional school or a lower educational attainment (6% missing values). Diagnostic interviews were conducted either in a face-to-face setting or via telephone [refer to Supplementary Table 1 (available online)]. In a subsample of the overall study sample, the questionnaire assessment was repeated approximately 2 months after the first assessment to allow the calculation of retest-reliability. After the diagnostic interview, participants received a link and were asked to complete a set of online questionnaires.

### Development of the Questionnaire

The Thought-Shape Fusion Body Questionnaire (TSF-B) was developed to assess cognitive distortions related to the imagination of thin ideals. The development of the questionnaire was based on the short version of the TSF trait questionnaire (Coelho et al., 2013). The short version was chosen, as it has been demonstrated to have the same unifactorial structure of the original version, with the advantage of reducing subject burden. For the TSF-B, 26 items written in German were developed in accordance to the original three theoretically proposed aspects of TSF (feeling, moral and likelihood). The original items of the TSF questionnaire, which target the imagination of eating fattening/forbidden food, were replaced by items that refer to the imagination of thin ideals. The wording and structure of the items were maintained as much as possible, to align with the original version. For example: *likelihood* (e.g., “If I think about thin women, I want to check that my clothes aren’t fitting more tightly.”), *moral* (e.g., “Thinking about giving up my thin ideal is almost as immoral to me as actually giving it up.”) and *feeling* (e.g., “I feel fatter after picturing thin women.”). For the item reduction, items were checked for redundancy, so that items with similar content were compared, and the item with higher variance was chosen. This process results in a 12-item short version of the TSF *Concept* section. A bilingual person performed a translation from English to German, then an independent back translation (German to English) was performed and the results were checked for differences and adapted if necessary. All items were rated on a five-point Likert-scale ranging from 0 “not at all” to 4 “totally/always.” In addition to the *Concept* section, seven additional items were included to assess frequency, impact, suppression and uncontrollability of TSF-B related thoughts (*Clinical Impact* section, adapted from Coelho et al. (2013)). Refer to Supplementary Tables 3, 4 (available online) for all items of the TSF-B questionnaire.

### Measures

#### Diagnostic Interview (DIPS, Mini-DIPS)

Either the DIPS (Diagnostisches Interview für psychische Störungen; Schneider and Margraf, 2011), a structured diagnostic interview based on DSM-IV-TR, or its short version Mini-DIPS (Margraf, 2015) were used to assess mental disorders and to identify the HCs. The criteria for disorders were adapted according to the DSM-5 (APA, 2013). In previous studies applying the DIPS retest-reliability ranges from 0.35 (sleeping disorders) to 0.94 (substance-related disorders) (Cohen’s kappa) and the interrater reliability from 0.57 to 0.92 (Schneider and Margraf, 2011). For the Mini-DIPS comparable psychometric properties were found (Margraf, 2015). All interviews were conducted by trained raters (members of the research team or graduate students) and supervised regularly by one of the first and last author and a co-author (AW, SM, or EB).

#### Questionnaires

##### German version of the thought shape fusion trait questionnaire – adapted short version (TSF trait short German)

To assess eating- and body-related cognitions in response to the imagination of food cues, the short German version (unpublished, available from the authors) based on the short version of the TSF trait questionnaire (Coelho et al., 2013) was used. A back translation was performed to ensure accuracy of the translation. An individual who was bilingual in German and English translated the items to English, followed by an independent translation back to English. This version was checked for differences with the English original. In cases of discrepancies, the German wording was checked again and adapted if necessary. The resulting measure includes 13 items assessing the TSF concept (Concept section), while four items measure clinical impact (Clinical Impact section). Items are rated on a five-point Likert-scale ranging from “not at all” (0) to “totally/always” (4), except for two items, where a time specification was asked. The TSF trait demonstrated good psychometric properties and high reliability for the Concept section (Coelho et al., 2013). The Cronbach’s α for the TSF-short German 13-items scale in this sample was 0.96.

##### Eating disorder examination questionnaire (EDE-Q)

Eating-related psychopathology was assessed by 28 self-report items of the Eating Disorder Examination Questionnaire (EDE-Q; German version Hilbert and Tuschen-Caffier, 2006). Participants answered questions about the frequency and severity of ED behaviors and symptoms on a seven-point rating scale, ranging from 0 (not present) to 6 (present every day/extremely). Scoring yields a global score, as well as four subscales (restraint, eating concern, weight concern and shape concern). A Cronbach’s α of 0.97 for the global score and between 0.85 and 0.93 for the subscales was found (Hilbert et al., 2007). In the present sample Cronbach’s α of the global scale was 0.80.

##### Social attitudes toward appearance questionnaire (SATAQ)

To assess the sociocultural influence on body image, the Social Attitudes Toward Appearance Questionnaire (SATAQ; Heinberg et al., 1995) was utilized in its German version (SATAQ-G; Knauss et al., 2009). Using a five-point Likert scale (1–5), this 16-item questionnaire measures three subscales: perceived pressure, awareness and internalization of the media transmitted beauty ideals. For the German version, good internal consistency for the three subscales was reported (Knauss et al., 2009). The Cronbach’s α for the present sample was 0.93 for all items.

##### Body shape questionnaire (BSQ)/ Fragebogen zum Figurbewusstsein (FFB)

The Body Shape Questionnaire (BSQ) contains 34 items that refer to feelings about one’s own appearance and concerns about body shape in the last 4 weeks (Cooper et al., 1987). A short German version (Fragebogen zum Figurbewusstsein, FFB; Pook et al., 2002) with eight items was used. Each item is scored 1 (never) to 6 (always). The eight-item English short version showed excellent internal consistency (Evans and Dolan, 1993). For the present study using the German version, Cronbach’s α was 0.94.

##### Beck depression inventory (BDI-II)

The 21-item self-report *Beck Depression Inventory (BDI-II)* was employed to assess the severity of depressive symptoms during the previous 2 weeks (Beck et al., 1996). The German version (2009) was utilized. Under each item four options (ranging from “not present” to “severe”) are provided. The validity and reliability have been shown to be good (Beck et al., 1996; Hautzinger et al., 2009). The Cronbach’s α of the German version is ≥0.84 (Hautzinger et al., 2009). For the present sample a Cronbach’s α was 0.95 was obtained.

##### Screening-instrument rumination-suppression (RS-8)

The *RS-8* (Pjanic et al., 2013) is an eight item screening instrument to assess two maladaptive emotion regulation strategies (rumination and suppression), related to negative mood and depression. In this study only the *rumination* subscale was used. The items were rated on a scale from 1 (not at all) to 6 (totally). The rumination scale of the RS-8 revealed good reliability in non-clinical and clinical samples (Cronbach’s α > 0.77) (Pjanic et al., 2013). In the present sample Cronbach’s α was 0.91.

#### Body Mass Index (BMI)

The BMI was calculated by dividing the weight in kilograms by the square of height in meters in order to classify underweight and overweight as well as obesity. While a BMI under 18.5 displays significant underweight, a BMI of above 25 refers to overweight. Obesity is indicated by a BMI over 30 (WHO, 2015). BMI was calculated from self-reported weight and height.

### Statistical Analyses

For descriptive statistics and item analysis, we used the Statistical Package for Social Sciences (SPSS version 23). For exploratory factor analysis (EFA) and confirmatory factor analysis (CFA), we used the software package R (version 3.1.3, 2015, including the R package “lavaan”) (R Core Development Team, 2011; Rosseel, 2012).

In a first step, the data of both samples were included to reveal the factor structure of the TSF-B Concept section using exploratory factor analysis (EFA) with promax rotation. Promax rotation is an oblique type of rotation, i.e., factors are allowed to correlate. Oblique rotations are often preferred as they represent the more realistic situation in which latent constructs derived from questionnaires are expected to correlate rather than to be completely independent of each other. Thereafter confirmatory factor analysis (CFA) was conducted to assess the model fit of the factor structure as obtained from EFA. Since item values were measured on an ordinal scale ranging from 0 to 3 rather than being continuously distributed, we used the diagonally weighted least squares estimator with robust standard errors and mean and variance adjusted test statistic (WLSMV; Rhemtulla et al., 2012). To test for measurement invariance between the two groups (HC and ED) we assessed configural, weak, strong, and strict invariances. Configural invariance implies that the number of items and their loading on a particular factor is the same between the two groups. Weak invariance implies comparable loadings in magnitude, strong invariance implies both comparable loadings and item intercepts, and strict invariance implies comparable residual variances between the two groups as well as comparable loadings and item intercepts. Since all these models are nested within each other using increasingly restrictive parameter specifications between the two groups, they could be directly tested in a sequential manner, i.e., weak invariance against configural variance, strong invariance against weak invariance, etc.

To improve model fit, residual terms of the following item pairs were always allowed to covary: 1 vs. 2, 1 vs. 8, 1 vs. 12, 8 vs. 9, and 9 vs. 11.

Cronbach’s alpha was calculated to investigate internal consistency. Values of ≥0.70 were used as a criterion for satisfactory internal consistency. To test whether the TSF-B questionnaire differed in magnitude between the two diagnostic groups, a one way multifactorial analysis of variance (MANOVA) with the factor group (HC, ED) and the dependent variable the TSF-B trait subscales was conducted. Since preliminary analyses showed that the variables were not normally distributed, the relevant subscales were log-transformed. Analyses also showed a violation of the assumption of homogeneity of variance; therefore, the results of the Brown-Forsythe test, which provides good robustness, are reported. Bivariate and partial correlations were performed to examine convergent validity (Pearson’s correlation, with log-transformed variables). Significant levels were set at *p* < 0.05. Effects sizes (Cohens *eta^2^*) were calculated for MANOVA analyses.

## Results

### Sample Characteristics

Sample characteristics of the assessed variables are listed in **Table 1**. Except for BMI, the ED group showed significant differences in the mean scores on all variables compared to the HC group.

**Table 1:** Sample characteristics.

	HC (*N* = 317)	ED (*N* = 243)	Statistics	
	*M (SD*)	*M (SD*)	*F*	*p*^a^
Age	23.42 (5.06)	24.42 (5.79)	4.5	^∗^
BMI	21.61 (2.92)	21.59 (5.27)	<0.1	n.s.
BDI-II	4.21 (4.28)	20.63 (13.24)	429.4	^∗∗∗^
RS-8 Rumination	2.73 (1.11)	4.59 (1.20)	347.1	^∗∗∗^
EDE-Q Global	0.71 (0.67)	3.42 (1.39)	922.9	^∗∗∗^
TSF trait	4.02 (6.09)	21.74 (14.59)	381.0	^∗∗∗^
SATAQ total	43.19 (13.36)	58.53 (13.60)	178.4	^∗∗∗^
BSQ	16.12 (6.58)	33.03 (9.11)	602.1	^∗∗∗^

*HC, Healthy Control group; ED, Eating Disorder group; M, mean; SD, standard deviation; BMI, Body Mass Index; BDI-II, Beck Depression Inventory; RS-8, Screening-Instrument Rumination-Suppression; TSF trait, Thought-Shape Fusion trait questionnaire; EDE-Q, Eating Disorder Examination Questionnaire; SATAQ-G, Sociocultural Attitudes Toward Appearance Questionnaire; BSQ, Body Shape Questionnaire; ^∗^*p* < 0.05, ^∗∗^*p* < 0.01, ^∗∗∗^*p* < 0.001, n.s., non-significant. ^a^One way ANOVA*.

### Distribution of Items and Correlation Matrix

**Table 2** presents descriptives and item total correlation of all TSF-B items (Concept section) together (*N* = 560). Mean values varied between 0.75 and 1.70 and *SD* between 0.91 and 1.44.

**Table 2:** Mean (*M*), standard deviation (*SD*), and item-total correlation (*r*_it_) of the 12 items (Concept section; English translation).

Nr.	Item	*M*	*SD*	*r*_it_
**Subscale 1: Imagination of thin ideals**			
TSF-B 1	I feel fatter after picturing thin women.	1.70	1.44	0.77
TSF-B 2	If I think about thin women, I want to check that my clothes aren’t fitting more tightly.	0.76	1.22	0.74
TSF-B 8	I want to restrict my eating after imagining thin women.	1.30	1.40	0.80
TSF-B 9	The mere thought of thin women makes me want to exercise.	1.18	1.29	0.76
TSF-B 10	Just thinking about women that are thinner than me can actually make me look fatter.	0.81	1.32	0.76
**Subscale 2: Striving for own thin ideal**				
TSF-B 3	Thinking about giving up my thin ideal is almost as immoral to me as actually giving it up.	1.01	1.32	0.77
TSF-B 4	If I think about giving up my thin ideal this can really make me gain weight.	1.01	1.38	0.77
TSF-B 5	I feel huge if I just imagine not striving for my ideal weight for a month.	0.95	1.40	0.87
TSF-B 6	Thinking about giving up my thin ideal makes me want to check in the mirror that I don’t look any fatter.	0.81	1.27	0.78
TSF-B 7	Just thinking about not working toward my thin ideal for a month makes me want to cut down on what I eat.	0.91	0.91	0.91
TSF-B 11	Thinking about giving up my thin ideal makes me want to exercise.	0.97	1.31	0.75
TSF-B 12	I feel guilty if I just think about not working toward being thin any longer.	0.75	1.27	0.78

*M, Mean; *SD*, standard deviation; *r*_*it*_, item-total correlation*.

Inter-item correlations for the total sample are available in Supplementary Table 5. Correlations ranged between 0.55 and 0.81.

### Examination of the Factor Structure

Results from EFA conducted for the HC and the ED group separately suggested a two-factor solution for both groups, based on the number of eigenvalues greater than 1 [HC 5.0 (42%), 2.8 (23%); ED 4.4 (36%), 3.6 (30%)] and parallel analyses. In the total sample the two factors accounted for 79% of variance (in the HC sample 65%; in the ED sample 67%). CFA corroborated the two-factor solution for the total sample as well as when considering the HC and the ED group separately, as seen by the goodness-of-fit indices (**Table 3**). In contrast, a one-factor model, which was additionally conducted, fitted data comparatively worse, especially in women with EDs, but also in the HC group and in the total sample. Thus values for CFI were 0.994, 0.989, and 0.980, for RMSEA 0.072 (*p* < 0.001), 0.042 (*p* = 0.764), and 0.083 (*p* < .001), and for WRMR 0.929, 0.653, and 0.841 in the total sample, in HCs, and in women with EDs, respectively.

**Table 3:** Goodness-of-fit indices.

Model	Sample	MFTS	Df	CFI	RMSEA	*p*-value for RMSEA	WRMR
2 factors	Total	90.6	48	0.998	0.040	0.906	0.599
	HC	50.4	48	0.999	0.013	0.995	0.502
	ED	69.6	48	0.995	0.043	0.680	0.568

*MFTS, Minimum Function Test Statistic; Goodness-of-Fit Index; CFI, Comparative Fit Index; RMSEA, root mean square error of approximation; WRMR, Weighted Root Mean Square Residual*.

The standardized factor loading of the items in the two-factor model ranged from 0.71 to 0.87 for the HC group and from 0.73 to 0.92 for the ED group. Both subscales of the Concept section were highly correlated. Measurement invariance revealed no significant differences between the HC and the ED group with respect to configural, weak, strong, or strict invariances (*p* > 0.05 for each of the four tests for invariance between the two groups).

### Internal Consistency and Construct Validity

The coefficients for the internal consistency (Cronbach’s alpha) were 0.83 (subscale 1, *Imagination of thin ideals*), 0.82 (subscale 2, *Striving for own thin ideal*) and 0.95 (total scale) for the HC group and 0.90 (subscale 1), 0.86 (subscale 2) and 0.95 (total scale) for the ED group.

A one-way MANOVA was conducted to assess differences between groups in TSF-B scores, with the two subscales entered as dependent variables. Women in the ED group had higher TSF-B values on subscale 1 (*M* = 9.81, *SD* = 5.83) and subscale 2 (*M* = 12.66, *SD* = 8.22) than did women in the HC group (total scale: *M* = 4.61, *SD* = 5.84; subscale 1: *M* = 2.76, *SD* = 3.20; subscale 2: *M* = 1.85, *SD* = 3.20). In statistical tests, the group differences with log-transformed variables emerged as significant [subscale 1: *F*(1,558) = 272.21, *p* < .001, η^2^ = 0.33; subscale 2: *F*(1,558) = 516.34, *p* < 0.001, η^2^ = 0.48].

To assess convergent validity, the relationships between scores on the TSF-B subscales and on the questionnaires measuring general psychopathology (BDI-II), rumination (RS-8), ED pathology (EDE-Q), the original TSF questionnaire, thin ideal internalization (SATAQ-G), body dissatisfaction (BSQ) were examined. Moreover, the TSF-B Clinical Impact section was included. All correlations were highly significant (*p* < 0.01; see **Table 4**). An additional one-way MANOVA revealed that participants with a BMI < 18.5 had the highest values on TSF-B (underweight, *n* = 97; subscale 1: *M* = 6.94, *SD* = 6.66; subscale 2: *M* = 9.53, *SD* = 9.03), the second highest values were found for participants with a BMI above 25 (overweight, *n* = 81; subscale 1: *M* = 6.85, *SD* = 5.56; subscale 2: *M* = 7.32, *SD* = 8.01), and the lowest values for participants in the normal BMI range of 18.5–25 (normal weight, *n* = 378; subscale 1: *M* = 5.30, *SD* = 5.40; subscale 2: *M* = 5.61, *SD* = 7.49). Statistical tests were significant: subscale 1: *F*(2,553) = 3.44, *p* < 0.033, η^2^ = 0.012; subscale 2: *F*(2,553) = 9.60, *p* < 0.001, η^2^ = 0.034. When only including the ED sample, no differences between BMI subgroups were found [subscale 1: *F*(2,239) = 0.78, *p* < 0.461, η^2^ = .006; subscale 2: *F*(2,239) = 0.38, *p* < 0.686, η^2^ = 0.003].

**Table 4:** Pearson’s correlations between TSF-B subscales and other instruments in the HC group and in the ED group.

	TSF-B Subscale 1 (imagination of thin ideals)		TSF-B Subscale 2 (striving for own thin ideal)	
	*HC*	*ED*	*HC*	*ED*
TSF trait	*0.58*	*0.59*	*0.67*	*0.76*
EDE-Q _total_	*0.68*	*0.67*	*0.56*	*0.64*
SATAQ_total_	*0.59*	*0.67*	*0.34*	*0.54*
BSQ	*0.70*	*0.73*	*0.53*	*0.65*
*BDI-II*	*0.24*	*0.29*	*0.26*	*0.42*
*RS-8 Rumination*	*0.31*	*0.26*	*0.26*	*0.29*
*TSF-B Clinical Impact*	*0.65*	*0.71*	*0.60*	*0.68*

*HC, Healthy Control group; ED, Eating Disorder group. BDI-II, Beck Depression Inventory; RS-8, Screening-Instrument Rumination-Suppression; TSF trait, Thought-Shape Fusion trait questionnaire; EDE-Q, Eating Disorder Examination Questionnaire; SATAQ-G, Sociocultural Attitudes Toward Appearance Questionnaire; BSQ, Body Shape Questionnaire; TSF-B, Thought-Shape Fusion Body Questionnaire, TSF-B Clinical Impact, frequency, impact, suppression and uncontrollability of TSF-B related thoughts. All variables were log-transformed*.

As an indicator of the impact on an individual’s well-being and daily life, we used the Clinical Impact section of the questionnaire. As reported above, significant correlations of ≥0.60 with the TSF-B subscales were found. The ED group had much higher values on the TSF-B Clinical Impact section (*M* = 10.94, *SD* = 4.75) than did HCs (*M* = 3.15, *SD* = 2.93; *F*_1,379_ = 506.5, *p* < 0.001, *d* = 1.9). Moreover, the impact section assesses the time (in hours) per day spent with thoughts about one’s own slimness ideal. The ED group reported to be occupied with thoughts about their thin ideal on average 3.61 h per day (*SD* = 5.16), which was far more than in HCs (*M* = 0.50 h per day, *SD* = 0.79; *F*_1_,_248_ = 84.70, *p* < .001, *d* = 0.83).

To further investigate the specificity of the TSF-B questionnaire, partial correlations were used to explore the relationship between the TSF-B subscales and other measures, while controlling for depression (BDI-II) and a general tendency to ruminate (RS-8). When controlling for depressive symptoms, all correlations as reported in **Table 4** remained significant (*p* < 0.001, HC: *r* = 0.29–0.68; ED: *r* = 0.47–0.72). Similar results were obtained for rumination (*p* < 0.001, HC: *r* = 0.25–0.66; ED: *r* = 0.49–0.73).

### Retest Reliability

Retest reliability was assessed in a subgroup of *N* = 234 participants of the HC group. The timespan between the first and the second assessment was on average 10.4 weeks (*SD* = 4.22). As with the first assessment, Cronbach’s alpha was high for subscale 1 (Imagination of thin ideals; α = 0.87), subscale 2 (Striving for own thin ideal; α = 0.91) and the total scale (α = 0.93). The reliability coefficient (r_tt_) for subscale 1 was 0.77 and 0.85 for subscale 2.

## Discussion

The aim of this study was to examine the factor structure of the newly developed *Thought-Shape Fusion Body Questionnaire* (TSF-B) and to verify whether its psychometric properties differ between the HC and the ED group. To test whether TSF-B is associated with ED pathology, correlates of convergent validity were investigated. More pronounced levels of TSF-B were expected in individuals with higher ED pathology, depression, BD and pressure to conform to a thin ideal.

In line with previous studies examining the factorial structure of the original TSF questionnaire (Jáuregui Lobera et al., 2012; Coelho et al., 2013), data did not support the theoretical three-factor structure (likelihood, moral, and feeling) in the TSF-B questionnaire. Instead, our results found a two-factor solution to fit best for the Concept section of the TSF-B questionnaire. The first subscale was named *Imagination of thin ideals*, as it clusters five items that refer to the imagination of female thin ideals. The second subscale was named *Striving for own thin ideal*, as it contains seven items that assess thoughts about pursuing or abandoning attempts to reach the ideal.

In addition to these two subscales representing the *Concept* section of the TSF-B questionnaire, the *Clinical Impact* of TSF-B was assessed with seven items. These included self-reported impairment encompassing frequency, impact, importance of suppression, and uncontrollability of the TSF-B thoughts. The analyses showed that high scores on TSF-B are associated with considerable impairment and a significant amount of time spent with such thoughts in everyday life. The TSF-B Clinical Impact section seems to be particularly helpful within the clinical context, as it provides additional information beyond the presence or absence of distorted cognitions associated with TSF-B.

Our results confirmed that the TSF-B subscales clearly distinguish between HCs and individuals with ED symptoms. The present study demonstrates that body-related cognitive distortions (i.e., TSF-B) are associated to relevant factors in EDs such as thin ideal internalization, depressive symptoms, and rumination in both women with and without EDs. The high correlations of the TSF-B subscales with the feelings about one’s own appearance and concerns about own body shape (BSQ) and eating-related psychopathology (EDE-Q) were also found in previous research on the concept of TSF (Shafran et al., 1999; Jáuregui Lobera et al., 2012). In accordance with our expectations, TSF-B was associated with depressive symptoms and a tendency to ruminate, which supports the conceptualization of TSF as skewed and inflexible thinking and as a thought fusion (Trindade and Ferreira, 2014). These findings relate to core concepts of acceptance and commitment therapy (ACT), where the central problem of EDs has been conceptualized as the tendency of emotional avoidance, cognitive rigidity, as well as the fusion of thoughts with an experienced reality. Instead of challenging negative thoughts and the entanglement in negative cognitions, ACT interventions aim at increasing acceptance of skewed thinking and simultaneously engage in defusion from these thoughts, so that cognitions lose their translational effect on emotions and behavior (see e.g., Hayes and Pankey, 2002; Manlick et al., 2013).

Partial correlations (i.e., correlations controlling for the level of depressive symptoms and rumination tendency) still revealed significant positive correlations between TSF-B and severity of ED pathology, indicating a unique contribution of body-related cognitive distortions (TSF-B) in explaining eating-related pathology. Therefore, cognitive distortions associated with the imagination of the thin ideal seem to be at least partly independent from and do not represent a mere artifact of heightened tendencies for unspecific rumination or depressive symptoms. This finding underlines the specificity of TSF-B and its association with the severity of ED pathology. Similarly, it is important to consider that none of the interrelations between TSF-B and the before-mentioned constructs exceeded a value of.85 (Gau, 2011), which supports the assumption of distinct concepts. TSF in its original conception is thought to be closely connected to worries about (over)eating, whereas TSF-B represents a different process, changing self-perception by referring to a different external frame. The inner representation of the self may differ significantly from the objective body shape that is activated upon imagination of a thin ideal, as well as the mental representation of the ideal (which is often unrealistic).

Integrating cognitive distortions of the TSF-B type in the cognitive-behavioral model of ED pathology (Fairburn et al., 2009; Cooper and Fairburn, 2011), would allow further investigation of the potential behavioral consequences of the experience of TSF or TSF-B. For example, TSF may be associated with urges to restrict food intake, while TSF-B may be associated with body checking behaviors. To capture the effect of body-related cognitive distortions in daily life, outside of laboratory settings, it would be helpful to employ ecological momentary assessments (e.g., Leahey et al., 2011).

The present study has several limitations that warrant attention. The clinical group of women with EDs was of moderate size and consisted of threshold and subthreshold EDs. Due to an insufficient sample size (n = 93 for subthreshold EDs) it was not possible to analyze threshold and subthreshold EDs separately. Moreover, the course and/ or stage of treatment was not controlled for, which might influence the magnitude of TSF-B exhibited. The ED group included AN, BN, and BED diagnoses, which makes it a heterogeneous, although ecologically valid, group (e.g., in respect of BMI, level of control over eating, and presence of compensatory behaviors). However, *post hoc* analysis revealed no significant differences in TSF-B between bulimic and non-bulimic types of EDs, indicating similarities in cognitive distortions across different ED subtypes, which is in line with the transdiagnostic theory of EDs (Fairburn et al., 2003). In addition, results from analysis with BMI subgroups indicate that BMI is not a predictor of TSF-B scores in the ED sample, even if there were small effects of BMI in the whole sample (TSF-B values in the whole sample were higher for participants with underweight and overweight compared to participants in the normal BMI range). This suggests that TSF-B in the whole sample is not fully independent of current weight status (however, differences in TSF-B between BMI subgroups were small, whereas differences between the groups HC vs. ED were pronounced). Our findings in the ED sample are consistent with the transdiagnostic assumption of EDs, since BMI did not affect TSF-B. Nevertheless, in subsequent studies with larger sample sizes, diagnostic groups of EDs as well as groups with different comorbid disorders and disorder severity need to be differentiated to allow more specific statements about TSF-B. However, depressive symptoms were controlled for in the partial correlations, therefore it is unlikely that the high rates of simultaneous occurrence of depressive symptoms (one of the most frequent comorbid conditions in EDs) have affected the results (Coelho et al., 2013; Keski-Rahkonen and Mustelin, 2016).

The current study sample consisted of participants of different substudies across multiple sites, with multiple clinicians (>25) involved in interviews across sites, it was not feasible to assess interrater reliability of diagnostic interviews. All clinicians were trained in the administration of diagnostic interviews and received supervision on a regularly basis; therefore, the classification of participants into the respective groups (HCs versus participants with ED symptoms) is expected to be stable across sites. Another limitation is related to reliance on self-report to calculate BMI, since individuals who are obese or underweight might have erroneous reflections of personal weight and height (Keith et al., 2011). Even though the range of BMI was rather wide, we decided not to control for BMI, as self-evaluation is known to be more important in body image dissatisfaction and disordered eating patterns than actual BMI (Markey and Markey, 2005; Swami et al., 2010). Additionally, in order to identify TSF and TSF-B as a disorder-specific correlate, a group of patients diagnosed with mixed mental health diagnoses other than EDs should be included (Munsch, 2014). Moreover, the TSF-B concept has not been investigated in men so far, which would demand a gender specific adaption (Griffiths et al., 2016) taking into account distinct features of the male body ideal (Tylka, 2011). Adaption of the measure for use across the gender spectrum is currently underway.

In sum, the current study supports the existence of a specific cognitive distortion, TSF-B, revealing body-related skewed thoughts associated with the imagination of the thin ideal. On a theoretical level, TSF-B could represent another relevant factor in an extended sociocultural risk model of BD and ED pathology (e.g., Ferguson, 2013; see e.g., Leahey et al., 2011; Wyssen et al., 2016b). The TSF-B questionnaire is a valid tool to assess the concept of body-related distortions, with good internal validity, convergent validity and test–retest reliability. The clinical impact of TSF-B is evident with the increased frequency of body-related thoughts, rumination tendency, body concerns and eating pathology. Future validation attempts could consider behavioral consequences of TSF-B as well as the relation to psychophysiological measures, such as heart rate variability, when the distortion is active. More detailed investigations about the occurrence, stability and possible changes of TSF-B should follow. Longitudinal studies assessing TSF-B could evaluate the predictive value with respect to the onset or course of an ED, or to investigate changes in the cognitive distortion with and without treatment.

## Ethics Statement

The local ethic committees (Department of Psychology, University of Fribourg, ethics committee of the cantos Fribourg, Zürich, Thurgau, Aargau (Switzerland) and of the Ruhr-University Bochum (Germany) approved the study protocols of all studies from which data were included into the present analyses. This study was carried out in accordance with the recommendations of the mentioned ethics committees with written informed consent from all subjects. All subjects gave written informed consent in accordance with the Declaration of Helsinki. The protocol was approved by the mentioned ethics committees.

## Author Contributions

SM, AW, JC, and LD established the concept and adaption. LD, AW, SM, JC, and AM wrote up the first draft of the paper. NH, NM, EB, KS, JL, and SS were engaged in correcting and adapting the second version of the draft and recruited patients. ST (Private Clinic Aadorf), KW (Private Clinic Schützen Rheinfelden), BI (Center for Eating Disorders and Obesity Zofingen), and GM (University Hospital – Clinic for Psychiatry and Psychotherapy Zurich) participated in the recruitment of participants and read the final version of the manuscript.

## Conflict of Interest Statement

The authors declare that the research was conducted in the absence of any commercial or financial relationships that could be construed as a potential conflict of interest.
