# Supplementary material for: Cognitive Distortions Associated with Imagination of the Thin Ideal: Validation of the Thought-Shape Fusion Body Questionnaire (TSF-B)
Source: Front Psychol. 2017 Dec 19;8:2194. doi: 10.3389/fpsyg.2017.02194 (PMC5742168; doi:10.3389/fpsyg.2017.02194)
Supplement: Supplementary file 1 [file Table_1.DOCX]

**Supplementary Material – Table 1**

Table 1. Additional information on the included studies/ subsamples.

| Study | Sample size (n) and sample description | Drop-out | Diagnostic interview | Compensation for participation |
| --- | --- | --- | --- | --- |
| 00: TSF-B validation study; students at the Universities of Fribourg and Bern (Switzerland) | n=252 (healthy sample)^1^ | n=5 | Mini-DIPS (telephone) | course credits |
| 01: Patients at the center for psychotherapy at the University of Fribourg | n=48 (clinical sample) | n=1 | DIPS  (face-to-face) | none; data was part of the regular quality assessment of the center |
| 02: Pilot study of an experimental trial at the University of Fribourg | n=46 (healthy sample)^1^ | n=0 | Mini-DIPS (telephone) | course credits |
| 03: Healthy and clinical participants of an experimental trial at the University of Fribourg (see Munsch, 2014) | n=160 (mixed sample)^2^ | n=23 | DIPS  (face-to-face or telephone) | 250 Swiss francs or course credits |
| 04: Recruitment on online platforms addressing the topic of eating and weight related problems | n=23 (mixed sample) | n=2 | Mini-DIPS (telephone) | draw for 20 x 100 Swiss francs (optional participation) |
| 05: Participants of an online-survey study at the Ruhr-University Bochum recruited through flyers, posters, websites, Facebook, clinics, psychotherapists | n=64 (mixed sample) | n=2 | Mini-DIPS (telephone) | 30 Euro |

*Notes:* 1) Participants in this sample were originally recruited as healthy controls, however some appeared to fulfill the criteria of a diagnosis and were moved to the clinical sample for the analyses. 2) mixed = healthy controls and eating disorder patients; DIPS = Diagnostisches Interview für psychische Störungen; Mini-DIPS = short version. Total sample: N=560.
