# Supplementary material for: Cognitive Distortions Associated with Imagination of the Thin Ideal: Validation of the Thought-Shape Fusion Body Questionnaire (TSF-B)
Source: Front Psychol. 2017 Dec 19;8:2194. doi: 10.3389/fpsyg.2017.02194 (PMC5742168; doi:10.3389/fpsyg.2017.02194)
Supplement: Supplementary file 2 [file Table_2.docx]

**Supplementary Material – Table 2**

Table 2. Criteria for the diagnosis of a subthreshold eating disorder (ED).

| Diagnosis | Criteria |
| --- | --- |
| Subthreshold anorexia nervosa (AN) | All of the criteria for anorexia nervosa are met, except that despite significant weight loss, the individual’s weight is within or above the normal range |
| Subthreshold bulimia nervosa (BN) | All of the criteria for bulimia nervosa are met, except that binge eating and inappropriate compensatory behaviors occur on average less than once a week or less than three months (APA, 2013) |
| Subthreshold binge-eating disorder (BED) | All of the criteria for binge-eating disorder are met, except that the binge eating occurs, on average, less than once a week or less than three months (APA, 2013) |
| ED in partial remission | After full criteria for an ED were previously met, some, but not all, of the criteria have been met for a sustained period of time (for more specific remission criteria see, APA, 2013) |
| Diagnosis according to the EDE-Q items^1^ | *Subthreshold AN* according to the EDE-Q: BMI<18 and EDE-Q item 10>0 and EDE-Q item 11 or EDE-Q item 22 or EDE-Q item 23 or EDE-Q item 25 or EDE-Q item 26 >0  *Subthreshold BN* according to the EDE-Q: EDE-Q item 15>3 and EDE-Q item 16>3 or EDE-Q item 17 >3 or EDE-Q item 18>3 and EDE-Q item 22>0 or EDE-Q item 23>0 and AN=0  *Subthreshold BED* according to the EDE-Q: EDE-Q item 15 > 3 and EDE-Q item 16<3 and EDE-Q item 17<3 and EDE-Q item 18<3 and AN=0 |
| Diagnosis according to EDE-Q cut off^1^ | EDE-Q global score > 3.2 (according to Mond, Hay, Rodgers, Owen, & Beumont, 2004) |

*Notes:* 1) Participants did not met the diagnostic criteria for a disorder according to the diagnostic interview; however, in the ED specific questionnaire assessment (EDE-Q) they showed tendencies that refer to subthreshold overall ED pathology relying on the cut off.
