# Supplementary material for: Cognitive Distortions Associated with Imagination of the Thin Ideal: Validation of the Thought-Shape Fusion Body Questionnaire (TSF-B)
Source: Front Psychol. 2017 Dec 19;8:2194. doi: 10.3389/fpsyg.2017.02194 (PMC5742168; doi:10.3389/fpsyg.2017.02194)
Supplement: Supplementary file 3 [file Table_3.DOCX]

**Supplementary Material – Table 3**

Table 3. German TSF-B short version.

**Thought-Shape Fusion Body Questionnaire (TSF-B) – German version**

Bitte bewerten Sie jede der folgenden Aussagen, indem Sie das Feld ankreuzen, welches für Sie persönlich am besten beschreibt, wie sehr Sie der Aussage zustimmen oder wie wahr die Aussage für Sie ist (von *überhaupt nicht* bis *völlig/ immer*), auch wenn einige der Aussagen auf Sie irrational wirken. Bitte beantworten Sie jede Aussage ohne zu lange darüber nachzudenken.

***Section „Concept“***

|  | **Wie sehr stimmen Sie den folgenden Aussagen zu?** | **Überhaupt nicht** | **Ein wenig** | **Mässig** | **Sehr** | **Völlig/ Immer** |
| --- | --- | --- | --- | --- | --- | --- |
| 1. | Ich fühle mich dicker, nachdem ich mir dünne Frauen vorgestellt habe. | 0 | 1 | 2 | 3 | 4 |
| 2. | Wenn ich an dünne Frauen denke, will ich überprüfen, dass meine Kleidung nicht enger sitzt. | 0 | 1 | 2 | 3 | 4 |
| 3. | Darüber nachzudenken mein Schlankheitsideal aufzugeben, ist für mich moralisch fast genauso verwerflich, wie es tatsächlich zu tun. | 0 | 1 | 2 | 3 | 4 |
| 4. | Wenn ich darüber nachdenke, mein Schlankheitsideal aufzugeben, kann das dazu führen, dass ich wirklich zunehme. | 0 | 1 | 2 | 3 | 4 |
| 5. | Ich fühle mich enorm dick, wenn ich mir nur vorstelle, einen Monat lang nicht nach meinem Idealgewicht zu streben. | 0 | 1 | 2 | 3 | 4 |
| 6. | Wenn ich darüber nachdenke mein Schlankheitsideal aufzugeben, will ich im Spiegel überprüfen, dass ich nicht dicker aussehe. | 0 | 1 | 2 | 3 | 4 |
| 7. | Wenn ich nur daran denke, einen Monat lang nicht nach meinem Schlankheitsideal zu streben, will ich einschränken, was ich esse. | 0 | 1 | 2 | 3 | 4 |
| 8. | Ich will mein Essverhalten einschränken, nachdem ich mir dünne Frauen vorgestellt habe. | 0 | 1 | 2 | 3 | 4 |
| 9. | Allein der Gedanke an dünne Frauen führt dazu, dass ich körperlich aktiv sein will. | 0 | 1 | 2 | 3 | 4 |
| 10. | Allein der Gedanke an Frauen, die schlanker sind als ich, kann dazu führen, dass ich tatsächlich dicker aussehe. | 0 | 1 | 2 | 3 | 4 |
| 11. | Wenn ich darüber nachdenke mein Schlankheitsideal aufzugeben, will ich körperlich aktiv sein. | 0 | 1 | 2 | 3 | 4 |
| 12. | Ich fühle mich schuldig, wenn ich nur daran denke, nicht mehr danach zu streben, dünn zu sein. | 0 | 1 | 2 | 3 | 4 |

***Section „Clinical Impact“***

|  | **Wie sehr stimmen Sie den folgenden Aussagen zu?** | **Überhaupt nicht** | **Ein wenig** | **Mässig** | **Sehr** | **Völlig/ Immer** |
| --- | --- | --- | --- | --- | --- | --- |
| 15. | Wie oft haben Sie Gedanken an Ihr Schlankheitsideal? | 0 | 1 | 2 | 3 | 4 |
| 15a. | Wie viele Stunden pro Tag? |  |  |  |  |  |
| 15b. | Wie viele Tage pro Woche? |  |  |  |  |  |
| 16a. | In welchem Ausmass werden Sie im Allgemeinen von Gedanken an Ihr Schlankheitsideal beeinflusst? | 0 | 1 | 2 | 3 | 4 |
| 16b. | Wie sehr werden Sie durch Gedanken an Ihr Schlankheitsideal in Ihrem Alltag gestört? | 0 | 1 | 2 | 3 | 4 |
| 17. | Wenn Sie Gedanken an Ihr Schlankheitsideal haben, wie wichtig ist es dann für Sie, diese Gedanken wieder aus Ihrem Bewusstsein zu bekommen? | 0 | 1 | 2 | 3 | 4 |
| 18. | Wenn Sie Gedanken an Ihr Schlankheitsideal haben, wie schwierig ist es dann für Sie, diese Gedanken wieder aus Ihrem Bewusstsein zu bekommen? | 0 | 1 | 2 | 3 | 4 |
|  |  |  |  |  |  |  |
