# Supplementary material for: Cognitive Distortions Associated with Imagination of the Thin Ideal: Validation of the Thought-Shape Fusion Body Questionnaire (TSF-B)
Source: Front Psychol. 2017 Dec 19;8:2194. doi: 10.3389/fpsyg.2017.02194 (PMC5742168; doi:10.3389/fpsyg.2017.02194)
Supplement: Supplementary file 4 [file Table_4.DOCX]

**Supplementary Material – Table 4**

Table 4. English translation of the German TSF-B short version.

**Thought-Shape Fusion Body Questionnaire (TSF-B) – English translation**

Please rate each statement by putting a circle around the number that best describes how much you agree with the statement, or how much it is true of you (from *not at all* to *totally/ always*), even though some of your responses may seem irrational to you. Please answer every item without spending too much time on any particular item.

***Section „Concept“***

|  | **How much do you agree with the following statements?** | **Not at all** | **Slightly** | **Moderately** | **Very much** | **Totally/ always** |
| --- | --- | --- | --- | --- | --- | --- |
| 1. | I feel fatter after picturing thin women. | 0 | 1 | 2 | 3 | 4 |
| 2. | If I think about thin women, I want to check that my clothes aren’t fitting more tightly. | 0 | 1 | 2 | 3 | 4 |
| 3. | Thinking about giving up my thin ideal is almost as immoral to me as actually giving it up. | 0 | 1 | 2 | 3 | 4 |
| 4. | If I think about giving up my thin ideal this can really make me gain weight. | 0 | 1 | 2 | 3 | 4 |
| 5. | I feel huge if I just imagine not striving for my ideal weight for a month. | 0 | 1 | 2 | 3 | 4 |
| 6. | Thinking about giving up my thin ideal makes me want to check in the mirror that I don’t look any fatter. | 0 | 1 | 2 | 3 | 4 |
| 7. | Just thinking about not working towards my thin ideal for a month makes me want to cut down on what I eat. | 0 | 1 | 2 | 3 | 4 |
| 8. | I want to restrict my eating after imagining thin women. | 0 | 1 | 2 | 3 | 4 |
| 9. | The mere thought of thin women makes me want to exercise. | 0 | 1 | 2 | 3 | 4 |
| 10. | Just thinking about women that are thinner than me, can actually make me look fatter. | 0 | 1 | 2 | 3 | 4 |
| 11. | Thinking about giving up my thin ideal makes me want to exercise. | 0 | 1 | 2 | 3 | 4 |
| 12. | I feel guilty if I just think about not working towards being thin any longer. | 0 | 1 | 2 | 3 | 4 |

***Section „Clinical Impact“***

|  | **How much do you agree with the following statements?** | **Not at all** | **Slightly** | **Moderately** | **Very much** | **Totally/ always** |
| --- | --- | --- | --- | --- | --- | --- |
| 15. | How often do you think about your thin ideal? | 0 | 1 | 2 | 3 | 4 |
| 15a. | How many hours per day? |  |  |  |  |  |
| 15b. | How many days per week? |  |  |  |  |  |
| 16a. | Generally, to what extent do thoughts about your thin ideal affect you? | 0 | 1 | 2 | 3 | 4 |
| 16b. | To what extent do thoughts about your thin ideal interfere with your daily life? | 0 | 1 | 2 | 3 | 4 |
| 17. | When you have thoughts about your thin ideal, to what extent is it important for you to get them out of your mind? | 0 | 1 | 2 | 3 | 4 |
| 18. | When you have thoughts about your thin ideal, to what extent is it difficult to get them out of your mind? | 0 | 1 | 2 | 3 | 4 |
|  |  |  |  |  |  |  |
