# Supplementary material for: Cognitive Distortions Associated with Imagination of the Thin Ideal: Validation of the Thought-Shape Fusion Body Questionnaire (TSF-B)
Source: Front Psychol. 2017 Dec 19;8:2194. doi: 10.3389/fpsyg.2017.02194 (PMC5742168; doi:10.3389/fpsyg.2017.02194)
Supplement: Supplementary file 5 [file Table_5.DOCX]

**Supplementary Material – Table 5**

Table 5. Inter-item correlations for all TFS-B items.

|  | TSF-B 1 | TSF-B 2 | TSF-B 3 | TSF-B 4 | TSF-B 5 | TSF-B 6 | TSF-B 7 | TSF-B 8 | TSF-B 9 | TSF-B 10 | TSF-B 11 | TSF-B 12 |
| --- | --- | --- | --- | --- | --- | --- | --- | --- | --- | --- | --- | --- |
| TSF-B 1 | 1 | 0.64 | 0.57 | 0.61 | 0.62 | 0.55 | 0.58 | 0.75 | 0.64 | 0.62 | 0.51 | 0.53 |
| TSF-B 2 |  | 1 | 0.59 | 0.55 | 0.68 | 0.61 | 0.64 | 0.63 | 0.60 | 0.65 | 0.57 | 0.56 |
| TSF-B 3 |  |  | 1 | 0.63 | 0.71 | 0.61 | 0.69 | 0.58 | 0.55 | 0.57 | 0.61 | 0.69 |
| TSF-B 4 |  |  |  | 1 | 0.74 | 0.65 | 0.70 | 0.62 | 0.55 | 0.59 | 0.55 | 0.63 |
| TSF-B 5 |  |  |  |  | 1 | 0.70 | 0.81 | 0.65 | 0.62 | 0.64 | 0.68 | 0.75 |
| TSF-B 6 |  |  |  |  |  | 1 | 0.70 | 0.60 | 0.54 | 0.67 | 0.63 | 0.67 |
| TSF-B 7 |  |  |  |  |  |  | 1 | 0.65 | 0.61 | 0.62 | 0.71 | 0.75 |
| TSF-B 8 |  |  |  |  |  |  |  | 1 | 0.76 | 0.63 | 0.56 | 0.62 |
| TSF-B 9 |  |  |  |  |  |  |  |  | 1 | 0.59 | 0.67 | 0.55 |
| TSF-B 10 |  |  |  |  |  |  |  |  |  | 1 | 0.57 | 0.62 |
| TSF-B 11 |  |  |  |  |  |  |  |  |  |  | 1 | 0.63 |
| TSF-B 12 |  |  |  |  |  |  |  |  |  |  |  | 1 |
